# The need for intra aortic balloon pump support following open heart surgery: risk analysis and outcome

**DOI:** 10.1186/1749-8090-5-20

**Published:** 2010-04-05

**Authors:** Haralabos Parissis, Michael Leotsinidis, Mohammad Tauqeer Akbar, Efstratios Apostolakis, Dimitrios Dougenis

**Affiliations:** 1 Royal Victoria Hospital, Cardiothoracic Department, Grosvernor Rd, Belfast, Nothern Ireland; 2 Department of Statistics and Epidimiology, Patras University, Greece; 3 Cardiothoracic Department, Essex Cardiothoracic Centre, Essex, UK; 4 Department of Cardiothoracic Surgery Patras University, Greece

## Abstract

**Background:**

The early and intermediate outcome of patients requiring intraaortic balloon pump (IABP) was studied in a cohort of 2697 adult cardiac surgical patients.

**Methods:**

136 patients requiring IABP (5.04%) support analysed over a 4 year period. Prospective data collection, obtained.

**Results:**

The overall operative mortality was 35.3%. The "operation specific" mortality was higher on the Valve population.

The mortality (%) as per time of balloon insertion was: Preoperative 18.2, Intraopeartive 33.3, postoperative 58.3 (p < 0.05).

The incremental risk factors for death were: Female gender (Odds Ratio (OR) = 3.87 with Confidence Intervals (CI) = 1.3-11.6), Smoking (OR = 4.88, CI = 1.23- 19.37), Preoperative Creatinine>120 (OR = 3.3, CI = 1.14-9.7), Cross Clamp time>80 min (OR = 4.16, CI = 1.73-9.98) and IABP insertion postoperatively (OR = 19.19, CI = 3.16-116.47).

The incremental risk factors for the development of complications were: Poor EF (OR = 3.16, CI = 0.87-11.52), Euroscore >7 (OR = 2.99, CI = 1.14-7.88), history of PVD (OR = 4.99, CI = 1.32-18.86).

The 5 years survival was 79.2% for the CABG population and 71.5% for the valve group. (Hazard ratio = 1.78, CI = 0.92-3.46).

**Conclusions:**

IABP represents a safe option of supporting the failing heart. The need for IABP especially in a high risk Valve population is associated with early unfavourable outcome, however the positive mid term results further justify its use.

## Background

Intra-aortic balloon pump (IABP) is the most usable tool of temporary mechanical circulatory support for cardiac surgical patients suffered from low cardiac output in the early postoperative phase. Only in United States, more than 70.000 patients are supported annually by IABP [1,2]. Its beneficial action is attributed to a concomitant reduction in afterload of left ventricle with a substantial increase on coronary perfusion pressure due to an increased of aortic diastolic pressure [3,4].

The main indication of IABP use in cardiac surgical patients is peri-operatively in the treatment of a low cardiac output state refractory to the usual inotropic support. Furthermore, it has been used prior to surgery in patients having sustained mechanical complications following myocardial infarction, as well as in patients with refractory angina [5-7].

The hospital and also the 30-day mortality for the patients necessitating IABP is high because of the cardiac problems that led to the need for this pump, ranged from 26% to 50% [2,6,8].

Aim of this study was to analyse our clinical experience with IABP in a high risk cohort of operated patients. It includes a risk analysis by means of looking into variables predicting mortality and early adverse outcome. In addition, the 5-year survival was reported.

## Methods

Within a 4 year period between January 2000 and December 2004, 2697 consecutive adult patients underwent cardiac surgery; 136 patients (5.04%) required support with IABP. The mean age was 66.3 +/- 9.9 years (range from 39 to 82 years).

There were 99 (72.8%) males and 37 (27.2%) female patients. First operation was carried out in 119 patients (87.5%) and re-operations in 17 patients (12.5%). Brake down of the referrals showed elective 24.3%, urgent 50.7%, emergency 19.9% and salvaged operations in 5.1% of the cases. 16.9% of the patients were diabetics.

Data pertaining to the patients past medical history were studied and also variables (see Table 1) including age, gender, diabetes mellitus, hypertension, high cholesterol, smoking, history of peripheral vascular disease, BMI, preoperative NYHA classification, ejection fraction, history of previous myocardial infarction, serum creatinine, Euroscore, previous cardiac operations, indication and timing for IABP insertion, operative priority, the nature of the operation, cardiopulmonary bypass time and status following the procedure. The myocardial protection of choice was Blood cardioplegia solution delivered every 20 minutes in an antegrade fashion.

**Table 1:** The pre- and intra-operative data of the patients supported with an IABP.

General characteristics	
Number of patients	136

Male/female	99/37

Age (y/s)	66.3 ± 9.9

Height (cm)	171 ± 8

Weight (kg)	79 ± 10

BSA	1.77 ± 9.3

Hypertension	42 pts

Diabetes mellitus	24 pts

Euroscore	8.43 ± 4.5

Significant Left main CAD	17 pts

Ischemic mitral regurgitation 2+/4+	12 pts

Ejection fraction < 30%	49 pts

Operation's-time (min)	365 ± 52

Cardiopulmonary bypass-time (min):	
CABG	102.1 ± 34.72
AVR & CABG	161.5 ± 38.2
Complex Cases	205 ± 38

Myocardial ischemia-time (min)	89 ± 23

Post op Cardiac Index (L/min/m^2^)	2.4 ± 1.7

The indications for initiating treatment with IABP in this cohort of patients was the following: a) IABP support for persistent preoperative ischemia despite maximum medical treatment b) patients not able to be discontinued from CPB although forced inotropic support, c) patients in low-cardiac output status just after a "difficult" discontinuation of CPB, supported by high-doses of inotropes, d) patients with "difficult" discontinuation from CPB and spontaneous appearance of arrhythmia (premature ventricular beats or VT) not amenable in anti-arrhythmic continuous infusion and e) post cardiotomy low cardiac output syndrome. Prophylactic initiation of IABP treatment was not advocated in any of the cases. A Datascope system (Datascope Corp, Paramus, NJ) was utilised. The IABP was introduced percutaneously through the common femoral artery in 131 patients and through an open access of the femoral artery in the remaining 5 patients.

Correct placement of the device was routinely confirmed with Chest X Ray in ICU. Once mediastinal drainage was minimum (< 50 ml/h), patients were anticoagulated with Heparin infusion, keeping the ACT >180-200 sec. Routine administration of a Cefalosporin 2^nd ^generation in combination with vancomycin, through out the IABP support, was maintained.

### Statistical analysis

Collection of the data is served using the Patients Analysis and Tracking System (PATS) software. Eighty variables were prospectively collected and carefully validated before being analysed.

Categorical variables were tested using a qui square test or Fisher exact test (two-tailed), and continuous variables were tested using Students t test (two-tailed). A p Value of less than 0.05 was regarded as statistical significant. All calculations were made using SPSS 11 edition. Operative mortality is reported as 30 day mortality. Long term survival data were obtained by sending questionnaires to the medical practitioners (98.5% response). The median period of follow up was 64 ± 11 months. Survival analysis was performed according to Kaplan-Meier method using life tables. Survival rates were given as cumulative survival +/- standard error.

## Results

The CABG, Valve and CABG and Valve population requiring IABP consist off 58.8%, 10.3% and 16.2% of the total number of patients treated with an IABP.

The mean Euroscore of the patients requiring IABP was 8.43 ± 4.5 (range 4 to 16).

### Preoperative intraaortic balloon pump support

Twenty two patients underwent IABP support preoperatively (16.2%). There was one elective case due to intractable angina (4.5%) and 8(36.3%) urgent cases (operated on at the same hospital admission) due to angina refractory to medical treatment. Eleven cases (50%) were treated as an emergency and underwent an operation within 24 hours from the cardiology referral and 2 cases (9.2%) were in severe cardiogenic shock and were deemed salvaged.

Four patients died (mortality 18.18%) two from the emergency group & also the two patients operated on under salvaging conditions.

### Intra-operatively intraaortic balloon pump support

Intra-operatively, ninety patients (66.2%) needed intraaortic balloon inserted following failure to be weaned off cardiopulmonary bypass. The overall mortality for this subgroup was 33.33% (30 patients).

### Post-operatively intraaortic balloon pump support

Post operatively, twenty-four patients (17.6%) needed intraaortic balloon inserted due to low Cardiac output syndrome. The mortality of this subgroup was high, 58.33% (14 patients).

## Breaking down the procedures

The incidence of patients needed IABP support per year was between 4.2 and 5% with a mean incidence of 4.3 ± 0.6.

### The Coronary artery bypass graft (CABG) population

Out of 1919 CABG patients operated on (mean Euroscore 3.71 ± 1.25) over the same period (5% of those patients had an Ejection Fraction less than 30% with an overall mortality of 12.5%) eighty patients required IABP (4.17%).

Out of the entire subgroup requiring IABP, 3 patients underwent off pump CABG and 77 patients on pump. The mean CPB time was 102.1 ± 34.72 minutes.

The overall mortality of the subgroup requiring IABP was 16 patients (21.2%). There were 63 males (78.8%) and 17 females (21.2%). The mortality for the males was 14.28% and for the females 41.17% (p < 0.05). Nine patients requiring IABP support underwent a redo-CABG (11.25%) with a mortality of 11.1%.

### CABG and Valve population

Out of 211 CABG & Valve patients operated on over the same period, twenty two patients (10.42%) required support with IABP. 152 patients underwent CABG and AVR out of which 9 patients (5.92%) required IABP.

There were 53 GABG and MVR patients out of which 13 patients (24.5%) required IABP.

This subgroup consists of 13 males (59.1%) and 9 females (40.9%). The mean CPB time was 161.5 ± 38.2 min.

The overall mortality was 11 patients (50%). The mortality for the males was 53.84% and for the females 44. 44%.

### CABG and other

This group of patients consists of a high risk population of eleven patients. Six out of them underwent CABG & Ischemic Ventricular Septal Defect (VSD) repair with mortality of 50%.

### Valve population

Out of the total population of 281 AVR valves operated on during the study period, 7 patients (2.5%) required IABP. Out of the total population of 85 MVR valves, 4 patients (4.7%) required IABP. Out of the total population of 25 Double valves 3 patients (12%) required IABP. The overall mortality of the group was 9 patients (see Table 2). Although the mortality was high in this group of patients one has to state that the numbers reported are very small to derive conclusions.

**Table 2:** Procedures requiring IABP & mortality

Procedures	Number	Percent	Mortality
**CABG only**	**80**	**58.8**	**16 (21.2%)**

**CABG + Valve**	**22**	**16.2**	**11 (50%)**

**CABG + Other**	**11**	**8.1**	**6 (54.5%)**

CABG & VSD (6)			3

CABG & Lung Biopsy (1)			1

CABG & Aortotomy & Exploration LV (1)			1

CABG & LV Aneurysectomy (1)			0

CABG & Root Replacement (1)			0

CABG & SVG Aneurysm (1)			1

**Valve Only**	**14**	**10.3**	**9 (64.3%)**

**Valve + Other**	2	1.5	0

**Other**	7	5.1	6 (85.3%)

	**136**	**entries**	**48 patients**

### Redo-operations

Out of 136 patients requiring IABP support, 17 cases were redo-operations (12.5%). Nine patients have had redo CABG, three had AVR/MVR and CABG, one had CABG and Aortic root replacement, one had CABG and aneurysm on a previous saphenous vein, and two patients underwent second time MVR operations. The overall mortality for the group was 27.1%.

### Others

From all 11 patients with post infarction VSDs over the 4 year period, 6 patients died (Mortality 54.5%). In all these patients a preoperative IABP support had been applied.

Eight patients underwent pericardiectomy (without CPB) and 2 of them developed early postoperatively low cardiac output syndrome; they were supported with an IABP and died (mortality of 25%).

### Mortality & Morbidity

The overall 30 day mortality was 35.3%. The mortality was mainly due to a severe low cardiac output in 17 patients (12.5%), intractable sepsis 13 patients (9.6%)(MRSA 6, VRE 1, other 6), cardiac arrest 13 patients (9.6%), stroke 2 patients(1.5%), Ischeamic bowel 1(0.7%), Pancreatitis 1(0.7%), GI bleed 1 (0.7%).

A regression analysis (Table 3) taking into consideration all the variables mentioned at Materials and Methods, revealed that a female smoker with renal impairment who undergoes a complex lengthy procedure requiring IABP, has the higher mortality.

**Table 3:** Multivariate logistic regression analysis of the risk factors influencing mortality

		Status		O.R.	95% C.I.		
Risk factor		alive	dead				p value
Gender	male	65	32	1.00			
	female	20	15	3.87	1.30	11.6	0.015
							
Smoking	No	33	9	1.00			
	Yes	13	10	4.88	1.23	19.37	0.024
	Ex	39	28	3.62	1.20	10.98	0.023
							
Pre Op Creatinine	<=120	70	32	1.00			
	>120	15	15	3.33	1.14	9.70	0.027
							
Cross Clamp Time	<=80	66	22	1.00			
	>80	19	25	4.16	1.73	9.98	0.001
							
IABP	pre op	16	4	1.00			
	intra op	59	29	4.27	0.95	19.15	0.058
	post op	10	14	19.19	3.16	116.47	0.001

The incremental risk factors for development of complications were: Poor EF (OR = 3.16, CI = 0.87-11.52), Euroscore >7 (OR = 2.99, CI = 1.14-7.88), PVD (OR = 4.99, CI = 1.32-18.86).

The subgroup of patients required IABP support compare to the rest of the cardiac surgical population had a higher incidence of reoperation for bleeding (11.8% Vs 4.5%), prolong ventilation (42.6% Vs 7%), re-intubation rate (18.4% Vs 4.9%), tracheostomy rate (9.6% Vs 1.2%) and new dialysis required (23.5% Vs 4.9%).

### Follow up/Survival

**Figure 1:**
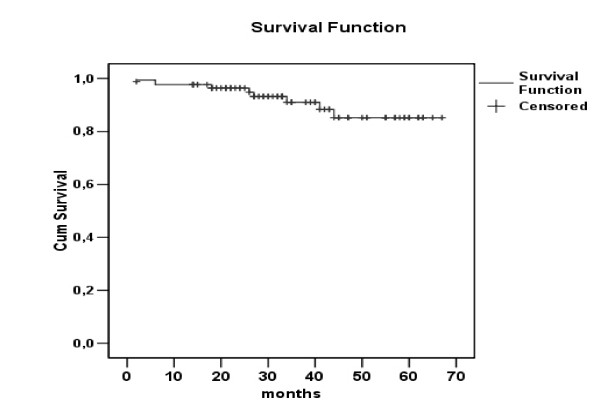
**Overall survival of the patients treated with an IABP**.

**Figure 2:**
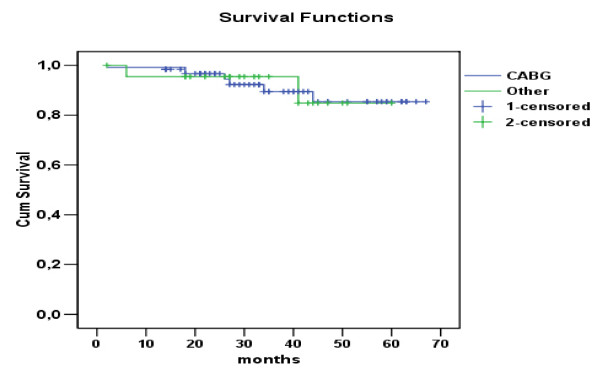
**Survival curves for the CABG group Vs Others**.

Actuarial survival curve for the entire group is presented in Figure 1. Cumulative survival for the entire group was 85.2% at 4 years. There was a difference in survival between GABG and Valve subgroups as per Figure 2. According to this the 5 years survival was 79.2% for the CABG versus 71.5% for the valve subgroup. (Hazard ratio = 1.78, CI = 0.92-3.46).

## Discussion

The need for increased use of IABP during cardiac surgery in the recent years has been reported by many groups [5,9]. This is mainly due to the fact that the patient population has changed and now includes older patients with multi-vessel disease and more impaired ventricles. On the other hand, there is a lower threshold for IABP use due to improve technology and lower rate of complications [5].

In our series IABP used in 5% of the cases, however its use was increased to 24.5% in patients requiring MVR and CABG procedure. This probably reflects the severity of LV dysfunction and the high incidence of low cardiac output syndrome in this group of patients.

As per other groups [10] the majority of the devices were inserted pre and intra operatively (82.4%). The preoperative indications were mainly unstable coronary syndrome with multivessel disease refractory to maximum medical therapy or symptomatic coronary disease with hemodynamic instability. IABP was not used for "prophylactic reasons"; it is unclear in the literature as to which patients would benefit from IABP support prior to surgery [11,12]. Some institutions however, they use the device too early and too often and they claim lower overall mortality [13].

The CPB time was prolonged (205 ± 38 min) for the complex cases. That was most probably due to: bleeding, a prolong "resting on CPB" after aortic cross-clamp removal because of difficulties in weaning from CPB and also a rather high threshold for intraoperative IABP insertion.

Ninety patients had intraaortic balloon inserted intraoperatively with a mortality of 33.3%. We attempt to split the intraoperative IABP insertion patient group into subgroups depending on time of IABP insertion and compare the outcome; however it became apparent that this was not feasible because the number of patients in those subgroups were too small to demonstrate any differences.

Through out the literature the mortality rates range widely from 7% to 86% [14,15]. This is probably due to the heterogeneous groups of patients considered. With the wide range of indications some series have included low risk patients, whereby the device was inserted prophylactically, with subsequent favourable outcome. The overall mortality in our series was around 36%. This obviously reflects a population of high risk patients. The mean age was high and also the percentage of patients operated on for a reason other than CABG was 41.2%. Comparing the overall mortality of the CABG patients needed the IABP device versus the entire CABG population with a poor EF we found that the first group has higher mortality 20% Vs 12.5%. Furthermore, higher mortality was detected (41.17%) in the female ischemic group that required treatment with IABP. The percentage of valve surgery patients requiring IABP is smaller (2.5%) compare to the CABG population. Therefore in our series out of a total number of 391 patients requiring single or double valve replacement (aortic ± mitral) 14 patients were supported with IABP. Nine patients died (64.3%). This is a group of patients with severe cardiogenic shock whereby the IABP was used post operatively with no real influence on the adverse outcome.

Timing of insertion and operative mortality has been reported by few groups [10,16] with outcome similar to our study. Like others [16] the lowest mortality was observed in elective male CABG patients to whom the IABP device had been inserted preoperatively. It is possible that better survival associated with preoperatively IABP insertion is predictable due to the fact that this subgroup is mainly suffer from intractable unstable angina in comparison to the subgroup requiring IABP support following peri or postoperative cardiogenic shock. Nevertheless, one would argue that optimal pre anaesthetic induction support with IABP minimizes perioperative ischemia and inotropic use and therefore reduces the incidence of postoperative cardiogenic shock. In summary, although this report failed to produce robust data, it showed a trend towards positive outcome when the IABP was inserted preoperatively.

Incremental risk factors for perioperative death have been reported by various investigators [10,17,18]. In a large retrospective study by Torchiana et al [17] independent predictors of death were age, MVR, prolonged CPB time, emergency operation, preoperative renal dysfunction, ventricular arrhythmias, right ventricular failure and emergency reinstitution of cardiopulmonary bypass. In another elegant study by Arafa et al [18] serum creatinine levels, EF, perioperative MI, timing of IABP insertion and indication for operation were independent predictors of early death. Although our study includes smaller number of patients the incremental risk factors for early death are similar with the aforementioned reports.

Surprisingly the overall mortality for redo CABG patients requiring IABP treatment was at around 11%. This is probably due to the fact that in the majority of those cases the EF was only moderate impaired and the IABP was inserted prophylactically preoperatively under stable circumstances.

The complication rates are higher in older studies [5,10,19,20] and lower in more recent publications [21-23]. In our study, IABP support was found to be associated with considerably higher morbidity, by means of prolonged Intensive Care Unit stay, CVVH support and tracheostomy rate. Those findings reflect the importance of multidisciplinary approach for providing care in this high risk subgroup.

In our report, cold pulse-less leg was detected in 1/4 of the cases. In 18 patients the ischemia resolved when the IABP was removed and in 8 patients following thrombectomy. Similar to other reports [21,24], poor EF and history of peripheral vascular disease were the incremental risk factors for development of vascular complications. In addition, Euroscore above 7 reflected the severity and comorbidity of the preoperative status of such patients.

Finally the cumulative survival of 85,2% in 4 years is rather higher compare with other groups[5,18,25]. Moreover there was a trend towards higher survival on the CABG population. (The 5 years survival was 79.2% for the CABG versus 71.5% for the valve group. (Hazard ratio = 1.78, CI = 0.92-3.46).

## Conclusions

This is a report of ongoing clinical practice. The subgroups (valves etc) of the patients supported with IABP are small; therefore the derived results should be taken with skepticism. The weaknesses of the study are due to its observational character; furthermore there may also be a selection bias for patients supported (ie.pre/postoperatively) with an IABP, due to individual clinical practices patterns. Lastly, variables that were not collected from the database (PATS) were obviously missed out from the multiple logistic regression analysis model.

In summary the peri-operative mortality of patients needed IABP support remains high. The mortality is increased exponentially when low cardiac output occurs in ischemic female population who also required concomitant valve surgery.

Nevertheless the use of IABP is justifiable. With respect to timing of IABP insertion, the literature is lacking on well defined guidelines. There is a trend to suggest that earlier use of the device is associated with better outcome possibly due to a better myocardial protection, but this remains to be tested with appropriate trials.

## Competing interests

The authors declare that they have no competing interests.

## Authors' contributions

Haralabos Parissis conceived of the study, gathered the data and wrote the manuscript, Michael Leotsinidis participated in the design of the study and performed the statistical analysis, Mohammad Tauqeer Akbar participated in the sequence alignment, Efstratios Apostolakis participated in the design and coordination Dimitrios Dougenis overlooked the progress of the manuscript and advised on valuable amendments. All authors read and approved the final manuscript.
